# The Anti-Inflammatory Effect of *Trichilia martiana* C. DC. in the Lipopolysaccharide- Stimulated Inflammatory Response in Macrophages and Airway Epithelial Cells and in LPS-Challenged Mice

**DOI:** 10.4014/jmb.2006.06042

**Published:** 2020-08-28

**Authors:** Ji-Won Park, Hyung Won Ryu, Hye In Ahn, Jae-Hong Min, Seong-Man Kim, Min-Gu Kim, Ok-Kyoung Kwon, Daseul Hwang, Soo-Yong Kim, Sangho Choi, Nelson Zamora, Kattia Rosales, Sei-Ryang Oh, Jae-Won Lee, Kyung-Seop Ahn

**Affiliations:** 1 Natural Medicine Research Center, Korea Research Institute of Bioscience and Biotechnology, Cheongju 286, Republic of Korea; 2 College of Pharmacy, Chungbuk National University, Cheongju 8160, Republic of Korea; 3 College of Pharmacy, Chungnam National University, Daejeon 414, Republic of Korea,; 4 International Biological Material Research Center, Korea Research Institute of Bioscience and Biotechnology, Daejeon 311, Republic of Korea; 5 Bioprospecting Research Unit, National Biodiversity Institute, Santo Domingo, Heredia 22-3100, Costa Rica

**Keywords:** *Trichilia martiana* C. DC, macrophage, inflammatory molecules, airway inflammation, NF-κB, acute lung injury

## Abstract

A number of species of the genus *Trichilia* (Meliaceae) exhibit anti-inflammatory effects. However, the effect of *Trichilia martiana* C. DC. (TM) on lipopolysaccharide (LPS)-induced inflammation has not, to the best of our knowledge, yet been determined. Therefore, in the present study, the antiinflammatory effect of TM on LPS-stimulated RAW264.7 macrophages was evaluated. The ethanol extract of TM (TMEE) significantly inhibited LPS-induced nitric oxide (NO), prostaglandin 2 (PGE_2_), inducible nitric oxide synthase (iNOS) and cyclooxygenase-2 (COX-2). TMEE also reduced the levels of inflammatory cytokines, including tumor necrosis factor-alpha (TNF-α), interleukin (IL)-1β and IL- 6. The upregulation of mitogen-activated protein kinases (MAPKs) and NF-κB activation was revealed to be downregulated following TMEE pretreatment. Furthermore, TMEE was indicated to lead to the nucleus translocation of nuclear factor erythroid-derived 2-related factor 2 (Nrf2) and the expression of heme oxygenase-1 (HO-1). In H292 airway epithelial cells, the pretreatment of TMEE significantly downregulated the production of LPS-stimulated IL-1β, and TMEE was indicated to increase the expression of HO-1. In animal models exhibiting LPS-induced acute lung injury (ALI), treatment with TMEE reduced the levels of macrophages influx and TNF-α production in the bronchoalveolar lavage fluid (BALF) of ALI mice. Additionally, TMEE significantly downregulated the activation of ERK, JNK and IκB, and upregulated the expression of HO-1 in the lungs of ALI mice. In conclusion, the results of the current study demonstrated that TMEE could exert a regulatory role in the prevention or treatment of the endotoxin-mediated inflammatory response.

## Introduction

Macrophage cells produce inflammatory molecules as a defense mechanism against bacterial infection. However, the uncontrolled activation of macrophage cells and the corresponding cell-derived inflammatory molecules can lead to hyper-inflammation, which causes acute inflammatory diseases, including acute lung injury (ALI) and sepsis [1, 2]. Inflammatory enzymes, including inducible nitric oxide synthase (iNOS) and cyclooxygenase-2 (COX-2) serve important roles during macrophage activation by inducing nitric oxide (NO) and prostaglandin E2 (PGE_2_) [3]. Macrophage-derived tumor necrosis factor-α (TNF-α), interleukin (IL)-1β and IL-6 have been indicated to induce alveolar inflammation and exacerbate lung damage [4, 5]. Airway epithelial cell-derived inflammatory cytokines, including IL-1β, have been demonstrated to serve an important role in the airway inflammatory response [6]. It has been well established that mitogen-activated protein kinases (MAPKs) and NF-κB are important in the regulation of the inflammatory response [2, 7]. Heme oxygenase-1 (HO-1) is critical for the amelioration of endotoxin-induced inflammation via the regulation of molecules associated with the inflammatory response [8].

Natural products have been indicated to exhibit beneficial effects for the prevention and treatment of inflammatorydiseases [9, 10]. *Trichilia* is a genus of the Meliaceae family, anda variety of species in this genus have been indicated to exhibit a variety of biological activities. The anti-inflammatory activity of *Trichilia silvatica* (C.DC) has been demonstrated in experimental models of arthritis [11], and *Trichilia catigua* A. Juss. has been indicated to ameliorate 2,4,6-trinitrobenzene sulfonic acid (TNBS)-induced colitis in an animal model [12]. However, to the best of our knowledge, the protective effect of *Trichilia martiana* C. DC (TM) in the lipopolysaccharide (LPS)-stimulated inflammatory response in vitro and in vivo has not yet been determined. In vitro study, the inhibitory effect of ethanol extract of TM (TMEE) on nitric oxide (NO), which is important marker in inflammatory response, was better than other species in LPS-induced RAW264.7 macrophages (Table 1). Therefore, in the present study, the beneficial effect of TMEE on the LPS-stimulated inflammatory response was assessed in RAW264.7 macrophages, H292 airway epithelial cells and mice.

## Materials and Methods

### Preparation of TMEE

*Trichilia martiana* C. DC. (TM) was collected at Carara National Park, Central Pacific Conservation Area, Costa Rica (Access resolution: R-CM-INBio-074-2009-OT issued by the National Authority, the Technical Office of the National Commission for Biodiversity Management, CONAGEBIO, Costa Rica) on May 2009 by the National Biodiversity Institute (INBio) and identified by Luis Diego Vargas, a plant taxonomic specialist of INBio. The voucher specimens (LDV-3772, KRIB 0028581) were deposited at CR and IBMRC herbaria. Dried and grinded samples of aerial parts of the plant were extracted three times using maceration that was assisted with an ultrasonic bath at room temperature with ethanol 95% for 30 min. The ethanolic extracts were separated using suction filtration via a Büchner funnel (Whatman grade 4V fluted filter paper, Cat No 1204-125). Solvent in combined filtrates was removed using a rotary evaporator, lyophilized and kept refrigerated at -20˚C until further use.

### Cell Culture

The RAW264.7 macrophages were purchased from the American Type Culture Collection (ATCC) and maintained in DMEM supplemented with 10% FBS (HyClone; GE Healthcare Life Sciences, USA) and a 1% antibiotic-antimycotic solution (Invitrogen; Thermo Fisher Scientific, Inc., USA) in 5% CO_2_ condition at 37°C. NCI-H292 airway epithelial cells were purchased from ATCC and maintained in RPMI 1640 medium (Gibco; Thermo Fisher Scientific, Inc., USA) supplemented with 10% FBS, in the presence of penicillin (100 U/ml), streptomycin (100 μg/ml) and 4-(2-hydroxyethyl)-1-piperazineethanesulfonic acid (HEPE, 25 mM), in 5% CO_2_ and 95% O_2_. To confirm the cytotoxicity of TMEE, a variety of TMEE concentrations were used. An MTT assay was used to examine the cell viability, as previously described (2, 13).

### Nitric Oxide (NO) and ELISA Assay

The concentration of nitrite in the supernatant was evaluated using the Griess reaction method [2]. The RAW264.7 cells were seeded (2.5 × 10^4^ cells) into 96-well plates and incubated with 0.5 μg/ml LPS (C-8274; Sigma-Aldrich; Merck KGaA, USA) in the presence of TMEE (5, 10, 20, and 40 μg/ml) for 20 h. A total of 100 μl cell culture supernatant was mixed with an equal volume of the Griess reagent and incubated for 10 min at room temperature. The absorbance at 540 nm was measured using a Spark 10 M multimode microplate reader (Tecan Group, Ltd.). The concentration of PGE_2_, TNF-α, IL-1β and IL-6 in the supernatant was investigated using ELISA kits, according to the manufacturer’s protocols. PGE_2_ concentration was determined using a PGE_2_ ELISA kit (Cayman Chemical Company). TNF-α, IL-1β, and IL-6 kits were purchased from R&D Systems, Inc., USA. The absorbance was measured at 450 nm using a Spark 10 M multimode microplate reader.

### Reverse Transcriptase (RT) PCR

The levels iNOS, COX-2, HO-1 and NQO1 mRNA expression were detected using RT-PCR. Isolation of the total RNA was performed using TRIzolTM reagent (Invitrogen; Thermo Fisher Scientific, Inc.), and cDNA was produced using a reverse transcription reaction, according to manufacturer’s protocol (Qiagen GmbH, Germany). PCR was performed using specific sense and antisense primers according to the manufacturer’s protocol (Bioneer Corporation, Korea). The amplification protocol was as follows: Denaturing at 94°C for 5 min (one cycle), followed by 94°C for 30 sec, annealing at 60°C for 30 sec and extension at 72°C for 45 sec (for 30 cycles), with a final extension performed at 72°C for 10 min. The primer sequences were as follow: iNOS forward, 5’¬CAAGAGTTTGACCAGAGGACC-3’ and reverse, 5’-TGGAACCACTCGTACTTGGGA-3’; COX-2 forward 5’¬GAAGTCTTTGGTCTGGTGCCTG-3’ and reverse, 5’-GTCTGCTGGTTTGGAATAGTTGC-3’; HO-1 forward, 5’-TGAAGGAGGCCACCAAGGAGG-3’ and reverse, 5’-AGAGGTCACCCAGGTAGCGGG-3’; NQO1 forward 5’-ACTACGCCATGAAGGAGGCT-3’ and reverse, 5’-TTCCAGCTTCTTGTGTTCGG-3’; and β-actin forward, 5’-TGTTTGAGACCTTCAACACC-3’ and reverse, 5’-CGCTCATTGCCGATAGTGAT-3’. β-actin was used as a housekeeping gene control. The products of PCR were separated by electrophoresis on a 1.5% agarose gel, and stained using ethidium bromide. The images were visualized using an Olympus C4000 Camera (Olympus Corporation, USA).

### Immunocytochemistry

RAW264.7 macrophages were seeded onto Permanox plastic chamber slides (Nalge Nulc International), and fixed with 4% paraformaldehyde at 4°C for 20 min. Triton X-100 (0.1%) was used in PBS to permeabilize the membrane, and 3% (w/v) BSA in PBS was usedto block the non-specific protein binding for 30 min. Subsequently, cells were incubated overnight at 4°C with an anti-NF-κB p65 subunit (rabbit polyclonal IgG; 1:200; Santa Cruz Biotechnology, Inc.) antibody. Cells were subsequently treated with an Alexa Fluor 488-conjugated secondary antibody (Invitrogen, USA; Thermo Fisher Scientific, Inc.) for 1 h at room temperature for nuclear staining, and were mounted using ProLong Gold Antifade reagent containing DAPI (Invitrogen; Thermo Fisher Scientific, Inc.) for 5 min. Images were captured using a confocal laser scanning microscope (LSM510m; Carl Zeiss AG, Germany).

### Isolation of Nuclear and Cytoplasmic Protein

Briefly, RAW264.7 cells were seeded (3 × 10^5^ cells) into 60 mm plates and incubated with 5, 10, 20, 40 μg/ml TMEE and 10 μM Bay11-7085 (Cat.no 14795, Cayman chemical) for 1 h. TMEE and Bay11-7085 were treated respectively or co-treated. After 1 h, LPS (0.5 μg/ml) was added to each well, and the plates were incubated for 1 h. Cells were harvested via mechanical scraping and split (Costar cell scrapers and lifters, USA) into nuclear and cytoplasmic fractions. Nuclear and cytoplasmic proteins were isolated using NucBuster protein Extraction kit (Cat, no 71183, Merck, Germany). Phosphate inhibitor cocktail tablets, 10mM sodium pyrophosphate (phosSTOP EASTpack, Roche, Germany) and the protease inhibitor cocktail tablets (39922700, Roche, Germany), were added to the CERI and NER extraction reagents prior to use.

### Western Blot Analysis

RAW264.7 cells were pretreated with TMEE for 1 h and stimulated with LPS. Cells were washed with PBS and lysed using lysis buffer (C-3228; Sigma-Aldrich; Merck KGaA, Germany). Lung tissues werehomogenized using a homogenizer in lysis reagent containing phosphatase and protease inhibitors (Roche Diagnostics, Switzland). The protein concentrations of each sample were determined using a Pierce BCA protein assay kit (Thermo Fisher Scientific, Inc.). The primary antibodies and dilution rates were as follows; Anti-phosphorylated (p)-c-Jun N-terminal kinase (4668), anti-p-p38 (9211), anti-p-ERK (9106), anti-p-NF-κB p65 (3033), anti-β-actin (4967; all, 1:1,000; all, Cell Signaling Technology, Inc.), anti-COX-2 (sc-1747), anti-JNK (sc-474), anti-p38 (sc-7149), anti-ERK (sc-154), anti-NF-κB p65 (sc-8242) anti-PCNA (sc-56; all, 1:1,000; all, Santa Cruz Biotechnology, Inc.), anti-iNOS (1:1,000; 905-431; Enzo Life Sciences, Inc.), p-IκB (15087), anti-HO-1 (1:1,000; 27338; all, Invitrogen; Thermo Fisher Scientific, Inc.) and anti-Nrf2 (1:1,000; 137550; Abcam, UK). Each protein was developed using a ECL detection system according to the manufacturer’s protocol (Thermo Fisher Scientific, Inc.). All bands were visualized using the LAS-4000 luminescent image analyzer (Fujifilm) and quantified by densitometry using Fuji Multi Gauge software version 3.0 (Fujifilm, Japan).

### Mouse LPS-Induced ALI Model

LPS-induced ALI was induced using the methods of Lee *et al* [14]. Healthy male C57BL/6N mice (*n* = 24; 6¬weeks old; body weight, 16-18 g) were purchased from Koatech Technology Corporation and were adapted to a specific pathogen-free condition (22-23°C; 55-60% humidity) with free access to food and water at least 1 week prior to the experiment. The oral administration of TMEE or dexamethasone (DEX) was administered for two consecutive days (on days 1-2). On day 2, mice were infected once intranasally with 40 μl PBS containing 10 μg LPS following the last oral administration of TMEE or dexamethasone (DEX). Mice were randomly divided into four groups as follows: Normal control group (NC), LPS (LPS administration) group, DEX (LPS administration + oral gavage of 1 mg/kg of DEX) group, and TMEE 10 (LPS administration + oral gavage of 10 mg/kg of TMEE) group. All animal experiments were approved by the Institutional Animal Care and Use Committee of the Korea Research Institute of Bioscience and Biotechnology (Korea) and performed in compliance with the National Institutes of Health Guidelines for the Care and Use of Laboratory Animals and the Korean National Laws for Animal Welfare.

### Measurement of Macrophage Numbers and TNF-α Production in the Bronchoalveolar Lavage Fluid (BALF)

To evaluate the regulatory effect of TMEE on macrophage recruitment and TNF-α production, BALF was collected as previously described (15). Mice were anesthetized using4 Zoletil 50 (30-50 mg/kg IP; Virbac Limited, France) and Xylazine (5-10 mg/kg IP; Bayer Korea, Ltd.) on day 3, based on prior anesthesia [16]. On day 3, mice received a tracheal infusion of ice-cold PBS (700 μl) twice (totalvolume, 1.4 ml), 24 h after the final administration of TMEE and DEX. To determine the macrophage number, 100 μl BALF was centrifuged on a glass slide at 264 ×*g* for 5 min at room temperature, and glass slides were stained using a Diff-Quik staining kit according the manufacturer’s protocol. Macrophage number was counted using a light microscope (magnification, ×400; scale bar 30 μm). The level of TNF-α in the BALF was determined using ELISA kits according to the manufacturer’s protocol (TNF-α ELISA kit; BioLegend, Inc., USA).

Statistical analysis. Values are presented as the mean ± standard deviation. Statistical significance was determined using a two-tailed Student’s *t*-test for comparisons between two groups. A one-way ANOVA followed by Dunnett’s multiple comparison test was used to analyze differences between multiple groups. Data were analyzed using SPSS 20.0 (IBM Corp., USA). *P *< 0.05 was considered to indicate a statistically significant result.

## Results

### Effects of TMEE on LPS-Stimulated NO, PGE_2_, iNOS and COX-2 in RAW264.7 Macrophages

**Fig. 1:**
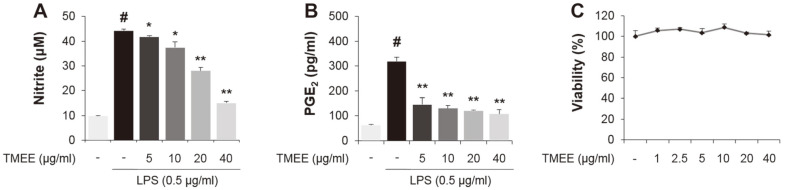
*Trichilia martiana* C. DC. ethanol extract (TMEE) reduces the release of NO and PGE_2_ in LPS-stimulated RAW 264.7 macrophages. The RAW264.7 cells were pretreated with TMEE (5, 10, 20, and 40 μg/ml) 1 h prior to incubation with LPS (0.5 μg/ml) for 20 h. The release of (**A**) NO and (**B**) PGE_2_ was examined using a NO assay and PGE_2_ ELISA kits. (**C**) No noticeable cell death was observed up to 40 μg/ml of TMEE. Data are expressed as the mean ± standard deviation. #*p* <0.05 vs. negative control group; *p < 0.05 and **p < 0.01 vs. LPS only group. TMEE, *Trichilia martiana* C. DC ethanol extract; NO, nitrous oxide; PGE_2_, prostaglandin-2; LPS, lipopolysaccharide.

**Fig. 2:**
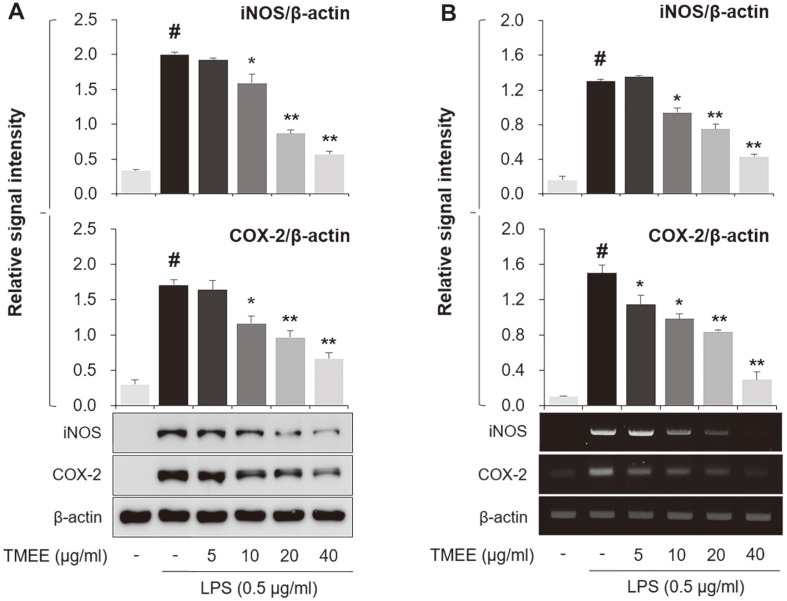
TMEE decreases the production of iNOS and COX-2 in LPS-stimulated RAW264.7 cells. >(**A**) The protein expression of iNOS and COX-2 were investigated using western blot analysis. (**B**) The mRNA expression of iNOS and COX-2 was determined using RT-PCR. Quantitative analysis of iNOS and COX-2 levels was performed using densitometric analysis. Data are expressed as the mean ± standard deviation. #P<0.05 vs. negative control group; *p < 0.05 and **p < 0.01 vs. LPS only group. TMEE, *Trichilia martiana* C. DC ethanol extract; iNOS, inducible nitric oxide synthase; COX-2, cyclooxygenase-2; LPS, lipopolysaccharide.

In the present study, the levels of NO and PGE_2_ were observed to be markedly increased in the supernatant of LPS-stimulated RAW264.7 macrophages (Figs. 1A and 1B). However, levels were decreased in a concentration-dependent manner by pretreatment with TMEE (5, 10, 20, and 40 μg/ml). The viability of RAW264.7 cells was not indicated to be markedly affected by the concentration of TMEE used in the experiment (Fig. 1C). The concentration set of TMEE was due to the MTT assay results reflecting up to concentrations where no significant cell death was observed. Based on the results of Fig. 1, the inhibitory effect of TMEE on iNOS and COX-2 was subsequently assessed by determining the levels of protein and mRNA in an in vitro experiment. As presented in Fig. 2, the treatment of LPS upregulated iNOS and COX-2 protein expression (Fig. 2A), which were also downregulated by the pretreatment of TMEE. To evaluate the regulatory effect of TMEE on the LPS-stimulated mRNA expression of iNOS and COX-2, RT-PCR was used. Supporting the results indicated in Fig. 2A, the increased mRNA expression of iNOS and COX-2 was significantly reduced by pretreatment with TMEE in LPS-stimulated RAW264.7 cells (Fig. 2B) (*p* < 0.01). These results demonstrated that TMEE exerts an anti-inflammatory effect in LPS-stimulated macrophages.

### Effects of TMEE on LPS-Stimulated TNF-α, IL-1β and IL-6 in RAW264.7 Macrophages

**Fig. 3:**
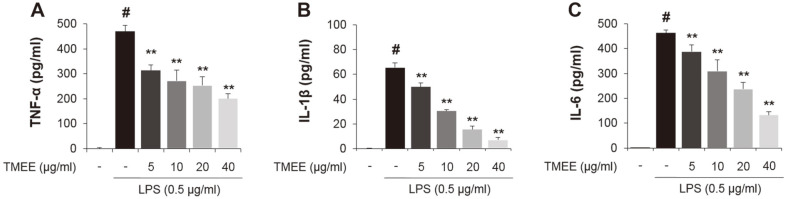
TMEE inhibits the release of (A) TNF-α, (B) IL-1β, and (C) IL-6 in LPS-stimulated RAW264.7 cells. The levels of TNF-α, IL-1β, and IL-6 were evaluated using an ELISA assay. Data are expressed as the mean ± standard deviation. #*p* < 0.05 vs. negative control group; **p < 0.01 vs. LPS only group. TMEE, Trichilia martiana C. DC ethanol extract; TNF-α, tumor necrosis factor-α; IL-1β, interleukin-1β; IL-6, interleukin-6; LPS, lipopolysaccharide.

The results of the current study showed the regulatory effects of TMEE on the upregulation of iNOS and COX¬2 (Fig. 2), and therefore, whether TMEE affected LPS-induced TNF-α, IL-1β, and IL-6 production was subsequently investigated. Culture medium supernatants were collected and the concentration of these molecules were examined using ELISA kits. As presented in Fig. 3, the increase of TNF-α, IL-1β, and IL-6 expression was confirmed using the administration of LPS. However, these results were significantly attenuated by pretreatment with TMEE in a concentration-dependent manner (*p* < 0.01). These results demonstrated that TMEE exerted an inhibitory effect on the production of pro-inflammatory mediators and a variety of cytokines.

### Effects of TMEE on LPS-Stimulated MAPK Activation in RAW264.7 Macrophages

**Fig. 4:**
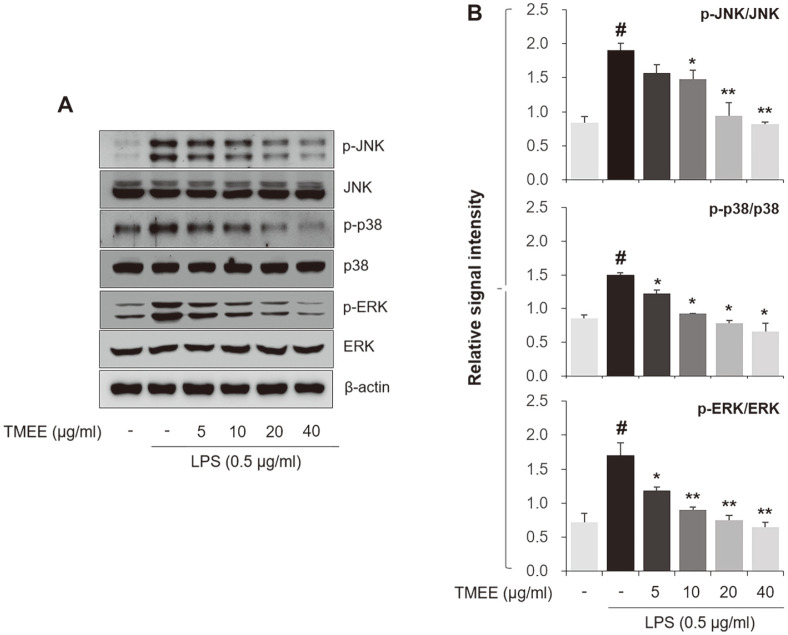
TMEE downregulates theactivationof MAPKs in LPS-stimulated RAW264.7 cells. (**A**) The levels of JNK, p38 and ERK phosphorylation was tested using western blot analysis. (**B**) Quantitative analysis of p-JNK, p-p38, and p-ERK levels was performed using densitometric analysis. Data are expressed as the mean ± standard deviation. #*p* < 0.05 vs. negative control group; *p < 0.05 and **p < 0.01 vs. LPS only group. TMEE, Trichilia martiana C. DC ethanol extract; MAPK, mitogen¬activated protein kinases; LPS, lipopolysaccharide; p, phosphorylated.

In the present study, TMEE was indicated to suppress LPS-induced inflammatory cytokines in RAW264.7 cells (Fig. 3). Therefore, whether TMEE regulates LPS-induced JNK, p38 and ERK activation was subsequently evaluated using western blot analysis. As presented in Fig. 4, the phosphorylated molecules were markedly upregulated by LPS treatment. However, pretreatment of TMEE was revealed to decrease these phosphorylated levels.

### Effects of TMEE on LPS-Stimulated NF-κB Activation in RAW264.7 Macrophages

**Fig. 5:**
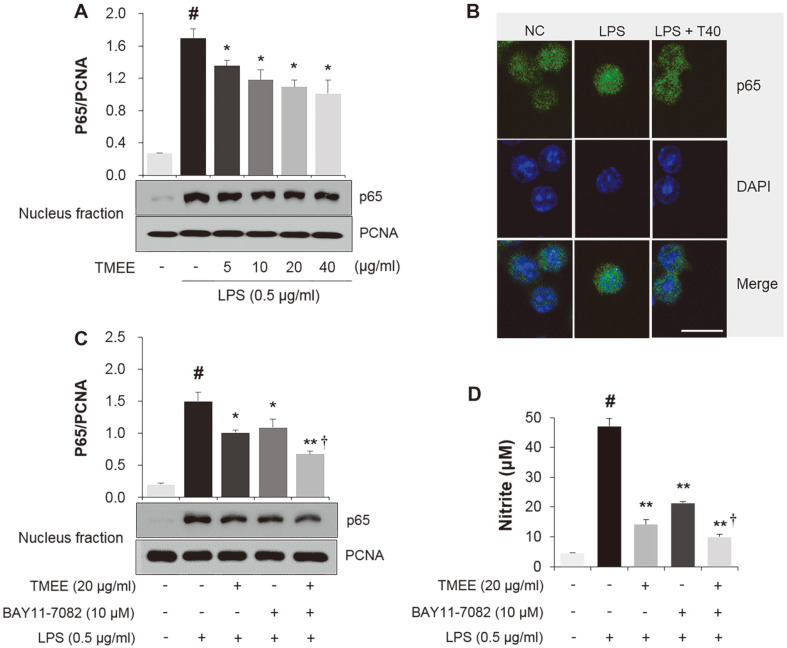
TMEE suppresses the nucleus translocation of NF-κB in LPS-stimulated RAW264.7 cells. (**A**) The protein level of NF-κB p65 in the nucleus was determined using western blot analysis. (**B**) The nuclear translocation of NF-κB was measured using immunocytochemistry. Cells were observed under a confocal microscope and images were captured using LSM 510 Meta software. Scale bar = 30 μm. The effects of a combination of TMEE and NF-κB inhibitor on (**C**) NF-κB nuclear translocation and (**D**) NO production in LPS-stimulated RAW264.7 cells. #*p* < 0.05 vs. negative control group; *p < 0.05 and **p < 0.01 vs. LPS only group; †*p* < 0.05 vs. TMEE only or BAY11-7085 group. PCNA, proliferating cell nuclear antigen. LPS + T40, LPS + TMEE 40 μg/ml; TMEE, *Trichilia martiana* C. DC ethanol extract; LPS, lipopolysaccharide; NO, nitrous oxide.

The regulatory effect of TMEE on LPS-induced NF-κB activation was subsequently assessed. Nucleus fraction was used to examine the inhibitory effect of TMEE on the LPS-induced nuclear translocation of NF-κB p65 in RAW 264.7 cells. The results showed that the levels of p65 protein were higher in the LPS-treated group compared with the NC group (Fig. 5A). However, this level was dose-dependently attenuated in the TMEE-treated group compared with the LPS-treated group. PCNA was used as an internal control. Immunocytochemistry revealed that the level of LPS-induced nucleus translocation of NF-κB p65 was also inhibited by pretreatment with TMEE (Fig. 5B). The inhibitory activity of 20 μg/ml TMEE on NF-κB p65 nucleus translocation was similar to that in 10 μM BAY11-7085, a NF-κB inhibitor (Fig. 5C). The combination of 20 μg/ml TMEE and 10 μM BAY11-7085 reduced the nucleus translocation of NF-κB p65 compared with the TMEE or BAY11-7085 treatment alone. Similar results were also indicated in the NO group (Fig. 5D), suggesting that TMEE attenuated LPS-induced NF¬κB nucleus translocation and NO production, and may be a valuable therapeutic inhibitor of NF-κB.

### Effects of TMEE on Nrf2 Nuclear Translocation and HO-1 Expression in RAW264.7 Macrophages

**Fig. 6:**
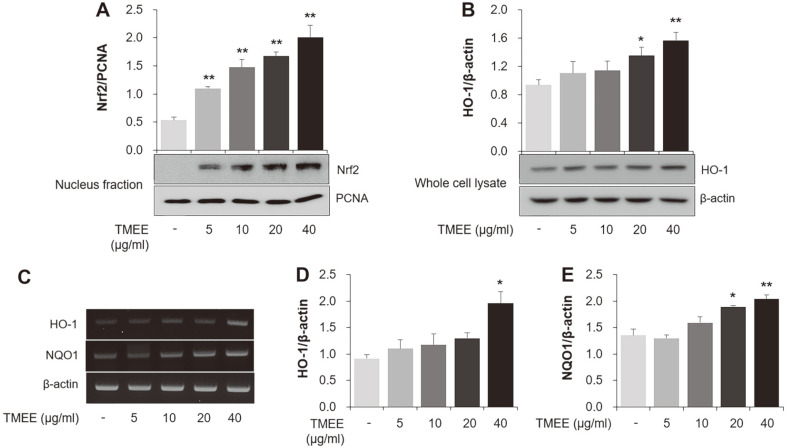
TMEE promotes the nuclear translocation of Nrf2 and the induction of HO-1 in RAW 264.7 cells. (**A**) The protein level of Nrf2 in nucleus was measured using western blot analysis. (**B**) The expression level of HO-1 in whole lysate was determined using western blot analysis. (**C**) The mRNA levels of HO-1 and NQO1 were determined using RT-PCR. Quantitative analysis of (**D**) HO-1 and (**E**) NQO1 levels was performed using densitometric analysis. Data are expressed as the mean ± standard deviation. *p < 0.05 and **p < 0.01 vs. negative control group. Nrf2, nuclear translocation of nuclear factor erythroid-2-related factor 2; TMEE, Trichilia martiana C. DC ethanol extract; HO-1, heme oxygenase-1; NQO1, NAD(P)H quinone oxidoreductase 1.

The nucleus levels of Nrf2 were measured using western blot analysis in the current study. As presented in Fig. 6A, thetreatmentof TMEE dose-dependently increased the expression of Nrf2 protein in thenucleusfraction lysate of RAW264.7 cells. The current study indicated that TMEE increased the expression of HO-1 in whole cell lysates of RAW264.7 cells (Fig. 6B). The mRNA expression of HO-1 was also revealed to be upregulated by TMEE treatment (Fig. 6C). Treatment of RAW264.7 macrophages with TMEE dose-dependently upregulated the mRNA levels of NQO1 (Fig. 6C). The ability of TMEE treatment to upregulate NQO1 mRNA supports the results of HO¬1 mRNA analysis.

### Effects of TMEE on LPS-Stimulated IL-1β and on the Expression of HO-1 in H292 Airway Epithelial Cells.

**Fig. 7:**
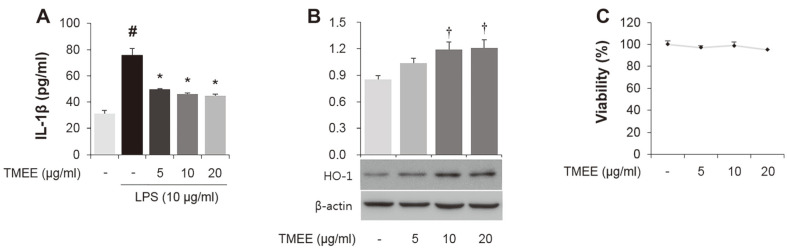
TMEE reduces the LPS-stimulated IL-1β and upregulates the expression of HO-1 in H292 cells. (**A**) H292 cells were pretreated with TMEE (5, 10 and 20 μg/ml) 1 h prior to incubation with LPS (10 μg/ml) for 20 h. The release of IL-1β was determined using ELISA kits. (**B**) H292 cells were treated with TMEE (5, 10, and 20 μg/ml) for 20 h. The expression level of HO-1 in whole lysate was examined using western blot analysis. (**C**) No noticeable cell death was observed up to 20 μg/ ml of TMEE treatment. Data are expressed as the mean ± standard deviation. #*p* < 0.05 vs. negative control group; *p < 0.05 vs. LPS only group. †*p* < 0.05 vs. negative control group. TMEE, *Trichilia martiana* C. DC ethanol extract; IL-1β, interleukin-1β; LPS, lipopolysaccharide; HO-1, heme oxygenase-1.

In the current study, whether TMEE leads to the downregulation of LPS-induced IL-1β in LPS-stimulated H292 cells was assessed. As presented in Fig. 7A, the significant increase in IL-1β was indicated in cells treated with 10 μg/ml LPS. However, this increase was significantly downregulated by pretreatment with TMEE (*p* < 0.05). TMEE pretreatment was also revealed to upregulate the expression of HO-1 in H292 cells (Fig. 7B). The concentration of TMEE (5, 10, and 20 μg/ml) did not affect cell viability in H292 cells (Fig. 7C).

### Effects of TMEE on Macrophage Influx and TNF-α Production in LPS-Induced ALI Mice

**Fig. 8:**
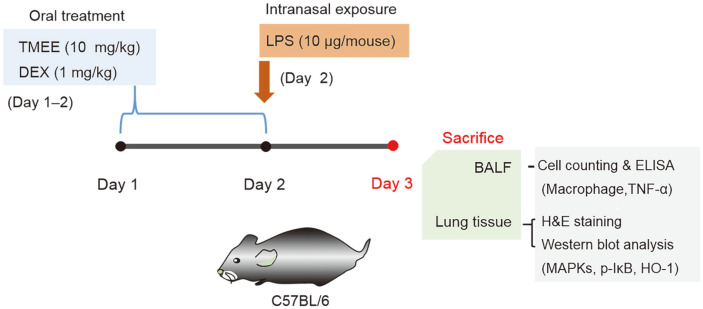
Experimental procedure for the pulmonary inflammation and administration of TMEE or dexamethasone (DEX). C57BL/6N mice were divided into four groups (*n* =6 in each group). The mice were orally administered TMEE (10 mg/kg) or DEX (1 mg/kg) from days 1 to 2. On day 2, the mice were given an intranasal administration of 10 μg LPS in 40 μl PBS 1 h after each corresponding TMEE or DEX administration. On day 3, the mice were sacrificed, and the BALF and lung tissues were harvested. TMEE, Trichilia martiana C. DC ethanol extract; LPS, lipopolysaccharide; BALF, bronchoalveolar lavage fluid; H&E, hematoxylin and eosin.

**Fig. 9:**
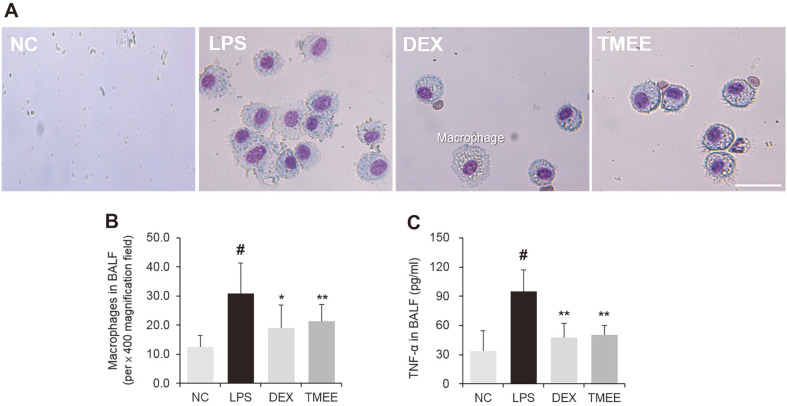
TMEE inhibits the recruitment of macrophages and the release of TNF-α in the lungs of LPS-induced ALI mice. (**A**) The microscope images of macrophages (magnification, x400; scale bar, 30 μm). (**B**) The count of macrophages.Diff-Quik® staining was used to determine the macrophages in the BALF.(**C**) The level of TNF-α in the BALFwas determined using a ELISA kit. Data are expressed as the mean ± standard deviation. #*p* < 0.05 vs. negative control group; *p < 0.05 and **p < 0.01 vs. LPS only group. TMEE, Trichilia martiana C. DC ethanol extract; TNF-α, tumor necrosis factor-α; LPS, lipopolysaccharide; ALI, acute lung injury; BALF, bronchoalveolar lavage fluid.

The sustained influx of macrophages is a characteristic of ALI, and leads to hyper-inflammation by inducing inflammatory molecules, including TNF-α [17]. Therefore, the regulation of macrophage recruitment and TNF-α production is an important therapeutic approach in ALI. Based on this result, the current study investigated whether TMEE affected macrophage recruitment in ALI mice. ALI was achieved by LPS administration (Fig. 8). The results of Diff Quik staining showed the numbers of macrophageswere increased in the BALF of LPS-induced ALI mice (Figs. 9A and 9B). However, treatment with TMEE reduced the LPS-induced increase of macrophages. The inhibitory rates of TMEE on macrophage numbers was 38.5% (DEX) and 31.2% (TMEE). As aforementioned, increased TNF-α expression is a major characteristic of ALI, and the inhibitory effect of TMEE on TNF-α in LPS-stimulatedRAW264.7 macrophages was previouslyindicated (Fig. 3). Therefore, the regulatory effect of TMEE on TNF-α in LPS-induced ALI mice was determined. As presented in Fig. 9C, the increase of TNF-α was demonstrated in the LPS-treated group, whereas treatment with TMEE significantly reduced this, suggesting that TMEE regulates LPS-induced macrophage influx and TNF-α expression (*p* < 0.01). The inhibitory rates of TMEE on TNF-α production was 49.87% (DEX) and 46.91% (TMEE). This inhibitory effect was similar to that of 1 mg/kg DEX, which was used as a positive control.

### Effects of TMEE on the Activation of MAPKs and IκB in LPS-Induced ALI Mice

**Fig. 10:**
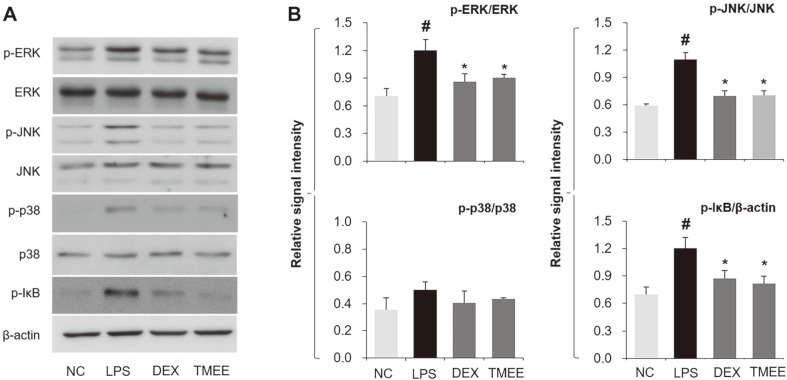
TMEE attenuated the activation of ERK, JNK and IκB phosphorylation in the lungs of ALI mice. (**A**) Levels of MAPKs and IκB activation were evaluated by western blot analysis. (**B**) Quantitative analysis of p-ERK, p-JNK, p-p38 and p-IκB was performed by densitometric analysis. Data are expressed as the means± standard deviation. #*P*<0.05 vs. negative control group; *p < 0.05 vs. LPS only group. TMEE, Trichilia martiana C. DC ethanol extract; ALI, acute lung injury; MAPK, mitogen-activated protein kinases; p, phosphorylated.

The levels of MAPK (ERK, JNK and p38) activation was demonstrated to be increased in the lung tissues of ALI mice (Fig. 10). ERK and JNK activation was attenuated in the TMEE treatment group and was supported by the results of the in vitro study (Fig. 4). However, LPS-induced p38 activation was not notably affected by TMEE treatment. It is well established that NF-κB activation is initiated by the activation of IκB, and blocking IκB activation reduces the inflammatory response by suppressing NF-κB nucleus translocation and inflammatory cytokines, including TNF-α (18, 19). The regulatory effect of TMEE on NF-κB nucleus translocation and TNF-α was demonstrated in the in vitro study (Figs. 3 and 5) and in the TNF-α in vivo study (Fig. 9). Therefore, it was expected that TMEE would suppress the LPS-induced IκB activation in LPS-induced ALI mice. As presented in Fig. 10A, the activation of IκB-α was indicated in the lung tissues of ALI mice, and this level was significantly downregulated following TMEE treatment (*p* < 0.05). The effect of TMEE on IκB-α inactivation was similar to the results of 1 mg/kg DEX administration.

### Effects of TMEE on the Induction of HO-1 in LPS-Induced ALI Mice

**Fig. 11:**
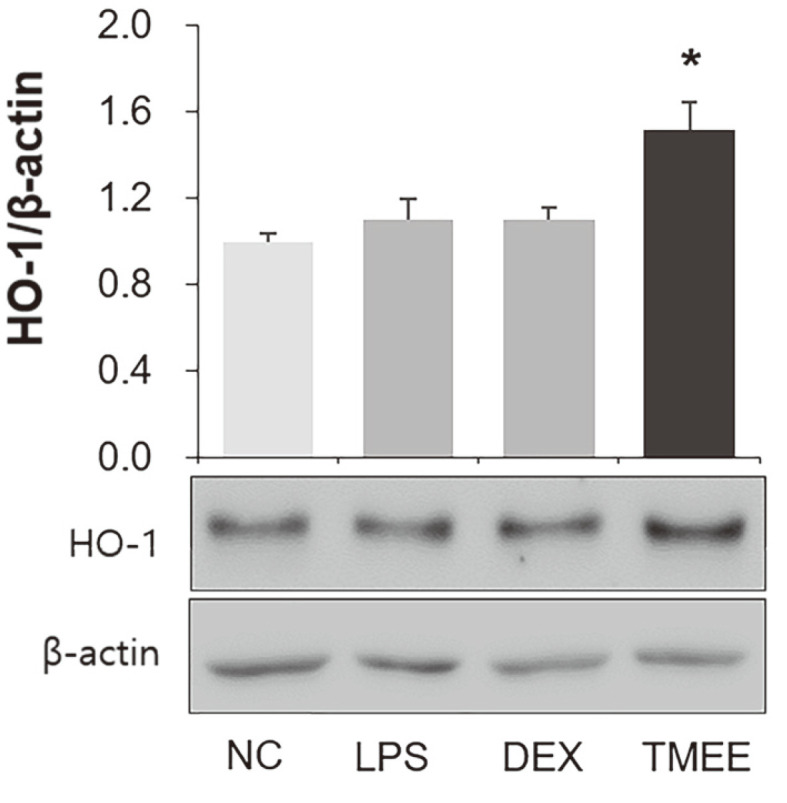
TMEE leads to upregulation of HO-1 expression in the lungs of ALI mice. Expression levels of HO-1 were examined by western blot analysis. Quantitative analysis of HO-1 was performed using densitometric analysis. Data are expressed as the mean ± standard deviation. *p < 0.05 vs. negative control group. TMEE, Trichilia martiana C. DC ethanol extract; HO-1, heme oxygenase-1; ALI, acute lung injury.

As aforementioned, the importance of HO-1 induction has been well established in previous studies, especially on inflammatory diseases, and the current study indicated that TMEE upregulated the expression of HO-1 in macrophages and airway epithelial cell lines (Figs. 6 and 7). Therefore, it was expected that TMEE can lead to the induction of HO-1 in ALI mice. The results of the present study demonstrated that treatment with TMEE significantly increased HO-1 expression in the lung tissues of ALI mice (Fig. 11) (*p* < 0.05).

## Discussion

The sustained production of inflammatory mediators and cytokines has been demonstrated to be associated with the pathogenesis of acute inflammatory diseases, including ALI and sepsis [5]. The high levels of macrophages influx and cell-derived inflammatory molecules, including NO, PGE_2_, TNF-α, IL-1β, and IL-6, have been indicated to cause excessive inflammation [2, 19, 20]. It was also reported that NO and PGE_2_ are important factors in the progression of ALI [21]. Endotoxin-induced inflammation has been revealed to lead to the upregulation of pro-inflammatory mediators, including iNOS, COX-2 and a variety of cytokines, including TNF¬α, IL-1β, and IL-6 [19, 22]. Therefore, regulating macrophage recruitment and inflammatory molecules is an important approach that can be used in the prevention or treatment of inflammatory diseases. In the present study, in vitro results showed that pretreatment of TMEE significantly decreased the levels of NO, PGE_2_, iNOS, and COX-2 in LPS-stimulated RAW264.7 macrophages (Figs. 1 and 2). The upregulation of TNF-α, IL-1β, and IL¬6 was attenuated by pretreatment of TMEE in a concentration-dependent manner (Fig. 3). In LPS-stimulated H292 cells, the significant increase in IL-1β was decreased by TMEE pretreatment (Fig. 7). In LPS-induced ALI mice, treatment with TMEE significantly reduced the levels of macrophages and the release of TNF-α (Fig. 9). These results indicated that TMEE exerted an ameliorative effect on LPS-induced macrophages influx and the release of inflammatory molecules.

The activation of JNK, p38 and ERK has been indicated to be upregulated in response to LPS and has been suggested to be closely associated with the production of inflammatory cytokines [23]. The production of inflammatory molecules, including NO, PGE_2_, TNF-α and IL-6 has been indicated to be initiated by the activation of NF-κB [14, 24]. Therefore, it has been widely accepted that the activation of MAPKs and NF-κB is associated with the pathogenesis of inflammatory diseases, and researchers have focused on the suppression of MAPKs and NF-κB activation [25, 26]. In the in vitro experiment performed in the current study, pretreatment of TMEE significantly suppressed the levels of JNK, p38, ERK phosphorylation and NF-κB p65 nucleus translocation in LPS-stimulated RAW264.7 macrophages (Figs. 4 and 5). Co-treatment with TMEE and BAY11-7085 was indicated to reduce the levels of NF-κB p65 nucleus translocation and NO production compared with TMEE or BAY11-7085 alone (Figs. 5C and 5D). In LPS-induced ALI mice, the upregulation of ERK, JNK and IκB activation was significantly reduced by treatment with TMEE (Fig. 10). These results suggested that the underlying molecular mechanisms of TMEE are closely associated with the downregulation of ERK, JNK and NF-κB activation.

Previous studies have demonstrated that the increase in HO-1 expression exerts anti-inflammatory effects by reducing inflammatory cytokines and chemokines in LPS-induced inflammatory diseases [27, 28]. It is also well known that HO-1 induction is associated with the downregulation of NF-κB activation [29]. Nucleus translocation of Nrf2 ameliorates the inflammatory response by inducing downstream antioxidant proteins, including HO-1 and NQO1 [30-33]. Airway epithelial cells release inflammatory cytokines, including IL-1β, which lead to a pulmonary inflammatory response by inducing macrophage recruitment [34]. In the in vitro experiment performed in the present study, TMEE treatment led to an increase in Nrf2 nucleus translocation, and HO-1 and NQO1 expression in RAW264.7 macrophages (Fig. 6). TMEE was also indicated to upregulate the expression of HO-1 in H292 airway epithelial cells (Fig. 7B). Furthermore, a significant increase in HO-1 expression was revealed in the lungs of ALI mice with TMEE treatment (Fig. 11). These results suggested that TMEE-induced HO-1 induction is closely associated with the amelioration of the LPS-induced inflammatory response.

Accumulating evidence has revealed that natural products exert ameliorative effects in inflammatory diseases, including in ALI [8, 19, 35]. These biological effects have been suggested to be closely associated with the suppression of macrophage influx and inflammatory mediators. Currently, the anti-inflammatory effects of TMEE have not been determined in vitro or in vivo. In the present study, TMEE was demonstrated to reduce LPS-induced inflammatory molecules in vitro. TMEE was also indicated to suppress the LPS-induced macrophages recruitment and TNF-α production in LPS-induced ALI mice. These effects of TMEE appear to be closely associated with the downregulation of MAPKs (especially ERK and JNK)/NF-κB signaling pathways and the upregulation of HO-1. In conclusion, the present study demonstrated the anti-inflammatory effects of TMEE and its possible mechanism, and revealed the potential for TMEE as a novel candidate for use in the prevention and treatment of inflammatory diseases, including ALI.

## Figures and Tables

**Table 1:** The inhibitory effect of the extract of several *Trichilia* species on nitric oxide (NO).

*Trichilia* species	Concentration	Inhibition rate of NO
*T. martiana* C. DC	40 μg/ml	51.30%
*T. elegans* A. Juss.	40 μg/ml	31.80%
*T. hirta* L.	40 μg/ml	30.20%
*T. glabra* L.	40 μg/ml	27.80%
*T. havanensis* Jacq.	40 μg/ml	23.10%
*T. pallida* Sw.	40 μg/ml	21.80%
